# Reviving Vavilov’s vision: The tragedy of biodiversity governance and principles for reform

**DOI:** 10.1073/pnas.2501753122

**Published:** 2025-12-12

**Authors:** David J. Bertioli, Soraya C. M. Leal-Bertioli, Tarikua Erda, Charles E. Simpson, Scott Barrett, Peter H. Raven

**Affiliations:** ^a^Institute of Plant Breeding, Genetics and Genomics, Center for Applied Genetic Technologies, University of Georgia, Athens, GA 30602; ^b^Department of Crop and Soil Sciences, University of Georgia, Athens, GA 30602; ^c^Department of Plant Pathology, University of Georgia, Athens, GA 30602; ^d^Leonard N. Stern School of Business, Finance Department, New York University, New York, NY 10012; ^e^Texas AgriLife Research, Department of Soil and Crop Sciences, Texas A&M University, Stephenville, TX 76401; ^f^School of International and Public Affairs, The Earth Institute, Columbia University, New York, NY 10027; ^g^Missouri Botanical Garden, St. Louis, MO 63110

**Keywords:** biodiversity, germplasm, governance

## Abstract

This perspective addresses two of humanity’s greatest challenges: feeding a growing population and conserving biodiversity. We begin by examining the legacy of Nikolai Vavilov, who pioneered the improvement of crops such as wheat and beans by hybridizing them with their wild relatives. This strategy used wild species biodiversity to introduce new genetic variation into crops, making them more resilient and productive. Its adoption around the world greatly increased food security and brought lasting benefits to humanity. However, since the 1990 s, well-intentioned laws shifted the governance of biodiversity from a shared global resource to the sovereign control of nation states, with serious unintended consequences. These changes have disrupted the collection, preservation, exchange, and use of biodiversity, all of which are central to Vavilov’s strategy for crop improvement and to biodiversity science more broadly. Efforts at reform have been frustrated as the issues became moralized, inhibiting the open dialogue needed for change. Using foundational concepts shared by science and good governance, we propose seven empirically grounded principles for reform, to help realign biodiversity governance with its intended aims. We then illustrate one possible framework—underpinned by global financing to protect biodiversity hotspots—that would align incentives and work in synergy with the principles to foster practical reform. The principles, together with frameworks that align incentives, would create the conditions for stronger biodiversity conservation and research, agricultural development, global food security, and all the associated benefits to humanity.

We cannot build peace on empty stomachs (1). Neither can we preserve the environment while hungry people are forced to use ecosystems unsustainably to survive. Despite historical increases in food production, 700 million people remain malnourished. With the global population projected to grow by another two billion before stabilizing (2, 3), the challenge of ensuring adequate food remains urgent. How can we feed the growing population while minimizing ecosystem damage? A major obstacle is that many crops have suffered strong population bottlenecks during domestication, reducing their genetic variability and constraining improvements in yield and resilience.

The Russian scientist Nikolai Vavilov was deeply influenced by his father’s childhood suffering during famine. Born at the turn of the 20th century, he dedicated his life to increasing agricultural production. In a groundbreaking insight, he observed that cultivated crops naturally interbreed and acquire valuable traits when grown alongside their wild relatives. Seeing this, he realized that crop productivity could be improved by systematically collecting crop wild relatives^*^ and strategically crossbreeding them. Since most crops are now grown far from where they originated (4), international collaboration and seed exchange are essential parts of Vavilov’s vision. Some notable examples of dependence on crops that originated in distant lands include the United States (soybean, wheat, and peanut), Brazil (soybean, sugarcane, and coffee), Sub-Saharan Africa (cassava, rice, and cacao), and Europe (potato, maize, and tomato). Vavilov embarked on more than 100 collecting expeditions to the cradles of agriculture around the world. He founded the largest seed bank of his time, a treasure trove of genetic diversity for crop improvement (5, 6).

Tragically, Vavilov’s life and work were cut short. In Stalin’s Russia, the false theory of inheritance of acquired characteristics gained political favor, and Vavilov’s support for Mendelian genetics was considered bourgeois. He was imprisoned and died in a Gulag. The persecution of scientists who would not adhere to political orthodoxy damaged Russian science and undermined agriculture, leading to one of the worst famines in human history. However, outside Russia, Vavilov’s strategy for improving crops flourished and provided a legacy of enhanced food production worldwide.

The authors of this perspective are deeply committed to the collection, research, preservation, and use of crop wild relatives. Charles Simpson has dedicated nearly half a century to studying the wild relatives of peanuts. On 28 expeditions to Argentina, Bolivia, Brazil, Ecuador, Paraguay, Peru, and Uruguay, he has reached some of the most inaccessible regions of these countries, sometimes at mortal peril. He pioneered the application of Vavilov’s strategy to improve the peanut crop, proving to breeders that productive cross-hybridization with related wild species is possible despite complex sexual incompatibilities.

Inspired by Charles Simpson’s foundational work, David Bertioli and Soraya Leal-Bertioli have dedicated over 25 y to harnessing the genetic diversity of wild peanut species from their South American home. Through work in South America and the United States, and international collaborations, they apply Vavilov’s strategy to introduce new pest and disease resistances into the peanut crop.

Peter Raven has had a long and varied career as a botanist and advocate for conservation and biodiversity. For 39 y, he led the Missouri Botanical Garden, transforming it into a globally recognized center for botanical research, education, and horticultural display, while receiving many honors for his contributions to botany and conservation.

Tarikua Erda is an applied economist whose research explores how research, development, and innovation may be affected by policies and international treaties. Scott Barrett is a pioneer in the economics of international cooperation. His early involvement in biodiversity treaty negotiations led to a lifelong commitment to studying how institutions can promote global cooperation, including for biodiversity conservation.

Between us, we have extensive first-hand experience collecting, working with, and using wild relatives of crops and other wild plant species, as well as expertise in biodiversity governance and international treaty design.

In this perspective, we briefly outline the achievements of Vavilov’s strategy in enhancing crop yields and its profound benefits for humanity. We also discuss how this work has been impacted by changes in biodiversity governance—defined here as the interplay of laws, beliefs, and attitudes that shape authority and decision-making. In a troubling echo of history in Vavilov’s time, we highlight how ideology is once again intruding into science. Finally, we propose a set of principles to guide the evaluation and reform of current practices along with one possible new framework that treats biodiversity as a global good––a resource both to be used and, with the support of global financing, conserved for the good of all.

## The Proven Benefits of Vavilov’s Vision

Despite Vavilov’s persecution and murder under Stalin’s regime, his pioneering strategy of using wild species in crop improvement gained increasing international recognition and became widespread by the 1950s.

A notable example of the success of Vavilov’s strategy is the control of stem rust in wheat, a fungal disease that devastated this crop for millennia. The ancient Romans feared it so deeply that they sacrificed red animals each spring hoping to ward it off. Centuries later, it was Vavilov’s strategy—the transfer of resistance from wild wheats via hybridization—that finally kept this ancient scourge in check from the 1960s almost until the turn of the millennium. At the International Maize and Wheat Improvement Center in Mexico, hybrids with wild wheats were distributed worldwide through their germplasm^†^ bank. This helped boost global wheat production and was especially beneficial for small-scale farmers who often do not use fungicides. Though new virulent strains have emerged in recent decades, resistance from wild relatives is once again proving vital in overcoming them. Overall, since 1960, wheat yields in developing nations have approximately tripled, with wild genetics playing a pivotal role in this gain (7–11). Contrary to common belief, modern wheat cultivars now contain more diversity in many traits than traditional landraces, a trend directly attributable to Nikolai Vavilov’s ingenious strategy of using wild species in crop improvement (12, 13). Indeed, wild traits introduced into wheat include resistance not only to stem rust, but also to other major diseases, leaf rust, yellow dwarf virus, wheat streak mosaic virus, root lesion nematode, and powdery mildew, as well as to insect pests like Hessian fly. Wild genetics have even increased grain protein. Together, these have made modern wheats more resilient, productive, and nutritious than their predecessors (14). The benefits of wild genetics extend well beyond wheat, to nearly every major crop. Below we outline a few more examples:

In rice, wild relatives have provided resistance to grassy stunt virus and other diseases, protecting millions of hectares in Asia. Hybrid rice with greater vigor, adaptability, and yield is produced using the genetics found in a wild rice. It now accounts for more than half the rice grown in China. In tomato, virtually all modern disease resistance derives from wild species, and genes from wild relatives have also increased yield, fruit quality, and resilience, transforming a once-fragile crop into one of the world’s most productive and widely grown vegetables, valued by home and commercial growers alike (14).

The impacts of Vavilov’s strategy in less-studied crops are not as well documented but are coming to light through the application of increasingly powerful genetic analysis and DNA sequencing. For example, a study of the genetics of cassava (*Manihot esculenta),* a crop originating in South America and now a crucial source of food in Africa, showed how it benefited from an early application of Vavilov’s strategy (15). In the 1930s, a wild relative, the Ceará rubber tree (*M. glaziovii*), endemic to Brazil, was hybridized with cassava. Its wild genetics spread across Africa, interbreeding with both elite varieties and landraces, bringing resistance to the damaging brown streak disease, higher yields, and improved nutritional value, thus improving food security for millions.

Another demonstration of Vavilov’s remarkable strategy comes from a study led by two of us. We tracked the genetic influence of hybrids created in the 1960s in North Carolina between peanut (*Arachis hypogaea*) and a related wild species from Bolivia (*Arachis cardenasii*) (16). Seeds from these hybrids were sent in the early 1980s to the International Crops Research Institute for the Semi-Arid Tropics (ICRISAT) in India, where Dr. Shyam Nigam, then head of peanut breeding, quickly recognized their potential and distributed them to research stations worldwide. They became integral to peanut improvement programs around the globe, providing resistance to the crop’s most damaging diseases: fungal leaf spots and rust. However, over time, the wild origin of these resistances was almost completely forgotten. Only through recent genetic profiling were we able to reveal the lasting genetic influence of this wild species—identifying it in 251 peanut varieties across 30 countries in Africa, Asia, Oceania, and the Americas. Data from eight field trials worldwide showed that, on average, peanut varieties with wild genetics yielded nearly twice as much as local varieties without such genetic material. The improved yields had a profound impact on families in many countries; for example, women in Mali reported increased income that allowed them to send their children to school. When we shared the widespread impact of these peanut hybrids with Dr. Nigam, he was delighted and reminisced: “I remember my early days at ICRISAT when I used to share breeding populations and germplasm lines in thousands with my collaborators in Asia, Africa, and Latin America. Crop improvement efforts, and agriculture, in general, in poor and developing countries benefited a great deal from such seed exchange.” However, he also commented that the same thing could not happen today, lamenting, “Seed collection and exchange has now become almost extinct.”—What changed between then and now?

## Changes in Biodiversity Governance, and Their Consequences

The changes Dr. Nigam lamented began in the 1980s, driven by concerns over illegitimate intellectual property claims. At this time, advances in DNA sequencing, together with contested interpretations of patent law, fueled a rush to lay claim to the human genome on a massive scale (17–19). Later, biopiracy—a term used to describe the illegitimate use of biodiversity or traditional knowledge, often involving patents—gained widespread media coverage, with reports of companies patenting biodiversity and traditional knowledge. The idea that the neem tree, turmeric, and basmati rice were being patented understandably fueled outrage.

These events politicized the collection and exchange of plant germplasm. Previously, such resources were generally regarded as humanity’s “common heritage”. However, they increasingly came to be seen as the rightful sovereign property of individual nations. This shift was formalized in the United Nations Convention on Biological Diversity, which placed germplasm under national sovereignty. Unfortunately, in a political climate concerned with exploitation, biopiracy, and control rather than collaboration, a cascade of changes followed, creating a new regime of biodiversity governance with significant unintended consequences. Here and throughout, we use “biodiversity governance” to mean the interplay of international agreements, national laws, administrative and societal norms, and beliefs that shape authority and decision-making.

Following the Convention’s ratification in 1993, nations enacted a complex patchwork of restrictive laws that broadly limited biological material exchange—even for temporary loans of herbarium specimens^‡^. The perception of germplasm changed from a public good to government property, profoundly affecting scientific research. Scientists working in biodiverse countries found their role shifting from research and conservation to regulatory compliance, with laws that, in effect, treated them as potential biopirates. Burdensome paperwork, severe restrictions on seed exchange, and the threat of personal fines or legal action for inadvertent violations hindered both national research and international collaboration (20).

Many scientists were disoriented and troubled by the profoundly negative consequences for botanical collection, research, and crop improvement. While the Convention formally acknowledged the distinct nature of agricultural germplasm, its approach created new barriers to germplasm exchange. In response, the International Treaty on Plant Genetic Resources for Food and Agriculture (commonly referred to as the Plant Treaty) was implemented in 2004. The Treaty established a Multilateral System, intended to facilitate access and benefit-sharing for key crops. While this marked an important step forward, in practice it has had limited impact. Important crops like soybean, sugarcane, oil palm, and peanut remained outside the Treaty’s scope (since they were not included in its Annex I), and coverage of wild relatives is uneven.

Even within its limited scope, the Treaty’s effects have been modest. Its Standard Material Transfer Agreement was intended to simplify exchanges, but in practice multiple layers of approval are often still needed. Excessive bureaucracy, a culture of control among governments, protectionism, and fear of liability among scientists have further hindered its operation. The incentives it provides are also uncertain and deferred to an undefined future, reducing the motivation to conserve or provide access to germplasm. The limited impact of the Plant Treaty illustrates how governance reflects not only laws and regulations but also the values, attitudes, and priorities of the societies that enforce them. Even if bureaucratic culture were more favorable to germplasm exchange, the Plant Treaty primarily governs the sharing of existing germplasm, leaving new collections constrained by restrictive laws. As Bjørnstad et al. note, the system is so flawed that opportunities for crucial crop improvements are being lost at a time when they are needed most (21).

Despite working in an increasingly politicized and restrictive atmosphere, scientists have at times joined forces to push back. One such effort was a 2011 letter endorsed by 181 Brazilian scientists and germplasm bank curators, sent to the Ministry of the Environment (*SI Appendix*). The letter described how the changes in governance had “created an atmosphere of uncertainty, insecurity, and fear,” making it increasingly difficult to carry out essential scientific work. They warned that “while this continues, as it already has for more than a decade, the ecosystems of our country continue to be devastated, with loss of species and even extinction.”

In 2018, another forthright letter, titled “When the Cure Kills,” was published in Science and signed by 172 scientists from 35 countries (22). The letter critiqued how restrictive governance, originally designed to prevent exploitation, was instead hindering biodiversity research and collaboration. It warned that “biodiversity research has seemingly become suspect in the minds of many regulatory bodies,” creating a chilling effect on scientific inquiry. National regulations, implemented in anticipation of commercial benefits, have “curtailed biodiversity research by in-country scientists as well as international collaboration.” The letter also noted that “in some cases, researchers have even been prosecuted,” further illustrating the climate of fear. The authors underscored the urgency of reform, highlighting that “biodiversity is vanishing and scarce talent is walking away from research.” Together, these letters highlight a troubling reality: governance changes driven by fears of exploitation have unintentionally undermined the collection and research needed to conserve biodiversity and ensure sustainable agricultural progress.

## Did the New Biodiversity Governance Help? Gene Patents and Biopiracy

The changes in biodiversity governance following the Convention were a reaction to concerns over illegitimate property claims, the rush to patent newly sequenced genes, and widespread outrage over biopiracy. To assess whether this new governance produced positive impacts, we examined the most prominent cases of gene patenting and biopiracy:

The key early controversies in gene patenting began in 1991 when Craig Venter, then at the NIH, led efforts to file *en masse* patent applications for short gene sequences (Expressed Sequence Tags, ESTs). The move provoked strong opposition, as critics pointed out that patenting naturally occurring genetic material was legally unsound and risked privatizing fundamental biological knowledge. Under pressure, the applications were withdrawn in 1994 (23). Later, Venter founded Celera Genomics and again announced that he would pursue patents on human gene sequences (24). However, facing widespread opposition and legal uncertainty, Celera shifted to a subscription model for commercial gene sequence access rather than patents (25). Eventually, in 2013, a challenge against Myriad Genetics, another company pursuing aggressive gene patent claims, reached the US Supreme Court, which ruled that naturally occurring gene sequences cannot be patented (Supreme Court opinion: 569 U.S. 576, 2013). This decision effectively closed the door on any type of sequence-then-patent strategy that would have led to the illegitimate privatization of life. Nevertheless, the ruling does leave some ambiguity: cDNA sequences, a synthetic mirror of genes, remain patentable, provided they are part of an invention that also meets the standard requirements of nonobviousness and demonstrated utility. This issue requires resolution. In the authors’ view, neither gene sequences nor their synthetic copies should be patentable. However, what is notable is that these matters are playing out essentially independently of the Convention.

Then we moved on to examining the most prominent cases of biopiracy in detail. These were simpler to interpret than the issue of patenting genes, generally following a pattern: sensational headlines, public outrage, and the withdrawal or collapse of illegitimate intellectual property claims. These cases heightened fears of biodiversity privatization, but their eventual resolution reveals something surprising: either pressure from the scientific community and the public led to the withdrawal of egregious claims, or existing patent laws overturned almost all of them. Again, this has played out without the intervention of the Convention.

The first case involves a patent granted in 1986 (US Patent No. PP5,751) for a particular cultivated clone of the hallucinogenic vine Ayahuasca (*Banisteriopsis caapi*) used in traditional ceremonies. This clone, which was given the name “Da Vine,” was found growing in an Amazonian garden. It was reportedly distinguishable from standard forms by a different flower color and some distinct vegetative traits. The patent covered only cuttings from this one plant, and was only valid in the United States. Although highly restricted and of no practical consequence in South America or anywhere outside the United States, it does fit the charge of biopiracy and had significant symbolic impact. Indigenous groups and environmental organizations protested, and the patent was challenged and revoked in 1999 by the U.S. Patent and Trademark Office. However, this decision was appealed, and the patent was reinstated in 2001—only to expire in 2003. This was the least satisfactory conclusion of the high-profile cases reviewed here. We believe that with further review, the patent would have been rejected. Nonetheless, the practical implications of the case were very limited, impacting cuttings from only one plant and only within the United States.

Our second case of biopiracy involves a patent granted in 1994 for fungicidal formulations based on the neem tree (*Azadirachta indica*; European Patent No. EP0436257B1). The patent caused widespread outrage and was challenged by a coalition of Indian NGOs and environmental activists who showed that neem’s fungicidal properties were already well-documented in traditional Indian agricultural practices. This led to the patent being revoked in 2000 (26). The third case arose in 1995, when a U.S. patent was granted for the use of turmeric (*Curcuma longa*) for wound dressings (US Patent No. 5,401,504). This prompted widespread protests, particularly in India, where turmeric had long been used in traditional medicine. India’s Council of Scientific and Industrial Research successfully challenged the patent by providing evidence of prior knowledge and use of turmeric to promote healing, leading to the patent’s revocation in 1997. Both these cases were resolved entirely within the U.S. patent system (27). Their practical consequences were negligible outside the jurisdictions of the patents, but their symbolic impact was significant, reinforcing global fears of biopiracy.

Another prominent case arose in 1997 when RiceTec Inc. obtained a US patent (No. 5,663,484) concerning certain hybrids of basmati rice. This led to public uproar, with the media incorrectly reporting that the company had patented traditional basmati itself (28). In fact, the patent covered specific hybrids and methods, not landraces. In any case, legal challenges led to the withdrawal of most claims in 2001, leaving the patent covering only three company-developed hybrids (29, 30). Similarly, in 1999, a U.S. company patented the Enola bean (US Patent No. 5,894,079), a yellow bean variety derived from traditional Mexican beans. This was indeed a textbook case of corporations seeking to patent a traditional landrace that they did little to develop. However, when the case was formally reviewed, it was found that the bean was not genetically distinct from existing landraces, and the patent was revoked in 2008 (31).

To further our search for positive impacts of biodiversity governance, we also investigated another case—not of biopiracy, but of a negotiated bioprospecting agreement. In 1991, Costa Rica signed an agreement with the pharmaceutical company Merck through its national biodiversity institute, INBio, that attracted international attention as a model of benefit-sharing that linked conservation to research and commercial use (32). Although often discussed together with the Convention, this agreement was independent from it. In any case, it was eventually terminated, and since then, INBio has faced severe funding and operational challenges (33). Though biodiversity can be valuable for medical discoveries, these benefits are too low to bring about much biodiversity conservation (34, 35). It is a commonly held idea that germplasm is a ready-made gold mine, but this is misleading. Its value is only potential, and can be realized only through long and costly processes of search, testing, development, and regulatory compliance. Because there is an enormous quantity of biodiversity from which to sample, and because many species share similar traits, the value inherent in any one species for the development of a new plant variety or pharmaceutical is generally modest. By extension, the value of conserving another hectare of habitat for research and discovery alone is small: Conservation cannot be effectively funded through free markets.

## Governance That Hinders Much More Than it Helps

These cases reveal a central weakness in the Convention’s justification: Preexisting patent laws generally addressed intellectual property abuses, without the need for its mechanisms. The outcries—sometimes justified, sometimes exaggerated—fueled a global push for controls that restricted research, collaboration, exchange, and conservation, yet the Convention itself did not resolve these cases. A potential criticism is that although patent law prevented overreach, it did not ensure monetary benefit-sharing. However, this overlooks two crucial points. First, it ignores the nonmonetary benefits of scientific research, such as improved crops developed using Vavilov’s strategy, as demonstrated earlier. Second, the reality is that the Convention has failed to generate significant monetary benefit—the first instance of shared monetary benefit through any related international mechanisms only occurred 26 y following ratification (36). That case occurred under the closely associated Plant Treaty, when Nunhems Netherlands B.V. made a payment to the Benefit-Sharing Fund^§^ after using germplasm to develop improved vegetable seeds (37).

**Fig. 1.**
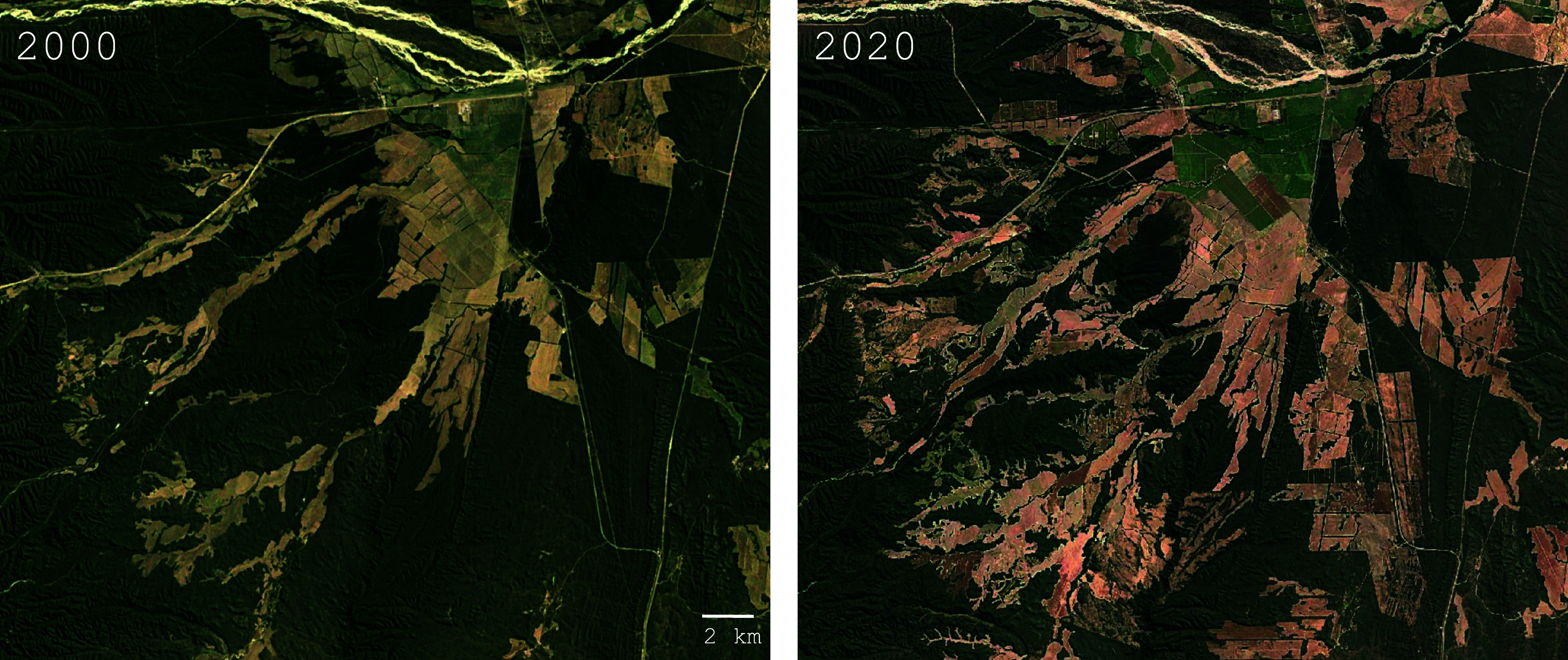
Satellite images from northwestern Argentina, about 35 km east of Salta, showing the expansion of agriculture near peanut’s center of origin during the 20 y that the Convention on Biological Diversity has been in force and new collection has been suppressed. This region is the native range of peanut’s A-genome donor, *Arachis duranensis*. During this period, many genetically unique populations of the species have been destroyed before they could be collected.

The Convention aspires to “the conservation of biological diversity, the sustainable use of its components, and the fair and equitable sharing of benefits arising from the utilization of genetic resources”—with particular emphasis on Indigenous Peoples and their traditional knowledge (38). Yet, in practice, it has not achieved these aims. Instead of fostering conservation, collaboration, and equitable benefit-sharing, it has restricted the collection, preservation, and exchange of crop wild relatives—all crucial prerequisites of scientific research and agricultural progress. Before the Convention, biological collections were widely shared, advancing knowledge and securing germplasm in multiple locations. Today, new collections are largely confined to germplasm banks within their country of origin, without duplication elsewhere, leaving them vulnerable to loss due to funding shortages or infrastructure failures. We are aware of multiple instances where unique germplasm has been lost due to these restrictions. Furthermore, in many countries, it is easier to obtain a permit to clear land for agriculture than to collect a few seeds or plant samples, accelerating habitat destruction while hindering conservation (Fig. 1).

This record of the new governance stands in sharp contrast to the well-documented and globally shared benefits of the historic model of free exchange, particularly through Vavilov’s strategy. While we previously cited a few notable examples, its full impact is difficult to quantify—once wild genetics are incorporated into a crop, they propagate and spread, often unnoticed by breeders. Studies attempting to quantify these contributions list hundreds to thousands of cases (14, 39), all (or nearly all) involving germplasm collected before the Convention’s restrictions took effect.

Though rooted in genuine fears of exploitation, the biopiracy controversies left a legacy not of productive reform, but of growing distrust in science and restrictive regulations that hinder biodiversity research and use. This disproportionately harms the poorest and most vulnerable, who stand to benefit most from agricultural progress. The Convention presents benefit-sharing as a path to equity, but in practice, it has not delivered tangible benefits to the powerless. The hungry gain more from food—unlocked by Vavilov’s collaborative vision—than from national ownership or monetary benefits that have never materialized. Bureaucracies that we now know deliver meager returns have become entrenched, while ecosystems fade and innovation stalls. Calls to expand the Convention’s restrictions to include DNA sequence data (40) would only compound these problems, further stifling research and limiting the potential for future agricultural innovations.

## Moralization and the Intrusion of Ideology into Governance and Science

How is it that these unintended consequences of the new biodiversity governance are not being widely recognized and discussed? How can we explain the strong emotions, opinions, and moral righteousness evoked by this subject, even among those with little or no direct experience or knowledge in this area? The answers must lie beyond any context of wild species, botanical collection, research, or regulations. We suggest they lie in human psychology and the dynamics of human societies. The language surrounding the Convention shifted away from the previously dominant ethos of collaboration to one centered on themes of the exploited and the exploiter. Pivoting around areas where discourse has become sensitive and constrained, it took on moralizing and ideological overtones. We have noticed during the writing of this manuscript that this section can provoke strong rejection, reactions that may themselves reflect the sensitized nature of this topic.

This sensitization and constraint around concepts such as nature, indigenous peoples, and sovereignty, can be understood as modern versions of sacralization which is a phenomenon of humans as a social species. All human societies display it, although it is directed in different ways in different societies. Sacralization goes beyond giving things their (in this case, enormous) due value and consideration; it involves a concerted emotional resonance within society so strong that the mere mention of the sacralized subject effectively prevents useful and critical discussion (41). Any person deviating from a consensus, emotionally resonant narrative risks being branded as a “bad person”, even when the narrative is plainly deficient. The resulting social sanctioning was identified by the 19th-century philosopher John Stuart Mill as the greatest impediment to a society’s ability to discern the truth (42). Competing viewpoints, values, and worldviews are unable to meet productively. Implementations using common sense and nuanced discussions of trade-offs become impossible, and empirical observations of real-world results are ignored.

At the dawn of the scientific era in the early 1600s, Sir Francis Bacon wrote on the importance of empiricism, the prioritization of practical observation over abstract ideas. A humorous parable that has been attributed to him (43) tells of a grievous quarrel in medieval times between brethren monks over the number of teeth in the mouth of a horse that extended for days. “All the ancient books and chronicles were fetched out, and wonderful and ponderous erudition such as was never before heard of in this region was made manifest”. Then, an innocent young monk plucked up courage and suggested they “look in the open mouth of a horse and find the answer to their questionings”. The brethren became enraged and “flew upon him and smote him, hip and thigh, and cast him out forthwith”. After days of more debate, they proclaimed that the answer to the question was an everlasting mystery. The parable highlights a persistent human tendency: to privilege textual authority and theoretical argument over direct empirical observation. Throughout history, deference to established narratives, in customs, religions, and laws, has often ostracized or punished those who seek answers through practical observation.

In the case of biodiversity governance, we see a similar pattern. Over the decades, hundreds of policy documents and academic articles have been developed, examining, and attempting to refine access and benefit-sharing mechanisms. Yet, empirical examination of outcomes remains strikingly rare. Looking (as it were) in “*the mouth of a horse*” reveals a clear picture of dysfunction, one widely recognized by germplasm curators and researchers worldwide. Extending the Convention’s restrictions to include DNA sequence data risks compounding these problems and would be disastrous. Ideological intrusion into the eminently practical problem of feeding the world is stifling Vavilov’s strategy, one of the most brilliant and impactful methods for increasing agricultural production and alleviating hunger. Vavilov’s own clash with Stalinist ideology resulted in his murder in a Gulag, but his legacy lived on. The clash of his ingenious strategy with the ideology associated with the Convention is more tragic because it involves food production for billions of people and germplasm that, once lost, can never be regained. We are confident that Vavilov would also regard it as more tragic because he undoubtedly saw his work as much bigger than himself. This is evident in his refusal to abandon Mendelian genetics even though he knew of the mortal peril of doing so (5, 6). It is also evident in the dedication of his colleagues who shared his mission; several of whom starved to death during the siege of Leningrad while faithfully guarding the seeds that they could have eaten.

In sum, botanical collecting, germplasm preservation, research, and the generation of benefits for humanity were far more effective when wild species were treated as a “common heritage” rather than restricted under the Convention and its associated governance. Appeals to speculative future gains and abstract ideals such as “biodiversity justice” ring hollow when compared to the tangible negative outcomes of these policies. This is not a rejection of moral concern; it is an appeal for governance that allows goals to be realized in practice. We believe a better path is possible, where biodiversity is better conserved and used, and humanity benefits.

## Principles for Reform

The main concern of the following section is not factual or scientific claims. It is not about what was, is, or will be. It concerns our aims and goals—what we decide ought to have been, or ought to be. In philosophical terms, this section is about norms, though here we will mostly use the more widely understood and broadly equivalent term “values”. As the Scottish philosopher of the Enlightenment David Hume emphasized more than two centuries ago, no logic bridges the gap between “is” and “ought”: what we value—our norms—are not facts outside our minds but matters of choice. Although no logic compels us to prefer one value over another, once chosen, values have logical consequences, and principles can be derived from them.

In the context of the present discussion, conserving biodiversity and benefiting humankind are primary values most readers will recognize as self-evident. Yet alternative worldviews do exist. One example is expressed in the phrase well-known in a significant strand of Amazonian politics: “my world is not of the jaguar and the monkey, my world is of men and cattle.”^¶^ This is not fringe; it is the mainstream worldview at Amazonian frontiers, with clear parallels across other biodiverse regions of the world. The failure to achieve our primary values of conservation and human benefit leaves our preferred worldview abandoned in practice, the space vacant for alternatives such as the men-and-cattle vision to occupy—all implemented too easily with different values, machetes, and fire.^#^

Ultimately, the failure to achieve our primary values is a question of governance, which for international biodiversity spans multiple nations, each with its own legal and institutional complexities. In such a system, isolated adjustments to individual rules or policies are unlikely to bring meaningful change. Only reform guided by fundamental principles can percolate through the network of international governance aligning its multiple components with its intended goals. Here we derive seven principles from our primary values of conserving biodiversity and benefiting humankind:

The first principle is based on our primary value of biodiversity’s intrinsic worth. The six subsequent principles are derived from our primary value of human benefit. Each is underpinned by an instrumental value—not an end in itself, but adopted because it reliably advances that goal. We chose these instrumental values based on the work of Robert Merton, Karl Popper, and Jonathan Rauch, who showed that they underpin both science and good governance—thus advancing human benefit. This is especially relevant to biodiversity governance, which not only regulates the use of natural resources but also shapes the scientific enterprise itself.

Robert Merton, the pioneering sociologist of science, in his seminal 1938 essay *Science and the Social Order* (44), showed how science depends upon norms shared with society. He later formalized these as—communalism, universalism, disinterestedness, and organized skepticism^||^ (45; often referred to by the acronym CUDOS). Karl Popper expanded on this theme, linking the ancient traditions of empiricism and critical discourse—both essential to the scientific process—with the principles of governance in open societies (46). More recently, public intellectual and scholar Jonathan Rauch wrote *The Constitution of Knowledge*, outlining how shared norms and procedures sustain scientific inquiry, legal systems, journalism, and democratic government, enabling the continued advancement of knowledge and society (47). The foundations of our principles, while articulated and championed by these scholars, are norms that originated in antiquity and were necessary preconditions for the development of science and good governance. They draw their legitimacy not from external imposition but from their proven efficacy.

These seven principles serve as practical guides for evaluating and improving biodiversity governance. They provide a structure to assess governance, to test whether its various components align with conservation and societal goals. Where governance falls short, adjustments are necessary to restore effectiveness and ensure it is fit for purpose.

## Principle 1: Preservation over Destruction

Governance that incentivizes and streamlines the conservation, collection, and sharing of biodiversity, ensuring that conservation is far easier than destruction or neglect, aligns with the Convention’s aims. When the destruction of habitat is easier than the collection of biodiversity, governance fundamentally contradicts the goals of the Convention.

### Foundation: Biodiversity has Intrinsic Value.

The Convention on Biological Diversity is founded on the norm that wild species have intrinsic worth and that their conservation serves a common good.^**^

## Principle 2: Simple over Complex Governance

Governance that is unnecessarily complex undermines the conservation of biodiversity. Complex bureaucracies create inefficiencies and often expand to perpetuate themselves, compounding their ineffectiveness. Simplification should be prioritized.

### Foundation: Ockham’s Razor.

This scientific norm, famously articulated by the 14th-century Franciscan friar and philosopher William of Ockham, holds that simple solutions should be favored over unnecessarily complex ones.

## Principle 3: Open Collaboration over Restrictive Control

Governance that makes sharing germplasm and information easier than withholding them promotes collaboration and conservation. If policies make withholding easier than sharing, they fail the test of serving biodiversity.

### Foundation: Merton’s Norm of Communalism.

The exchange of information and resources advances the collaboration and innovation that is integral to science and progress.

## Principle 4: Universal over Restricted Access

Governance that ensures universal access to germplasm for all stakeholders—regardless of nationality or affiliation—advances the goals of the Convention. Systems that favor access for some while restricting it for others hinder collaboration and progress.^††^

### Foundation: Merton’s Norm of Universalism.

Science and progress are best served by broad participation and fair opportunities for all contributors, regardless of background or affiliation.

## Principle 5: Impartiality over Vested Interests

Governance that prioritizes the common good—through the collection, preservation, research, use, and sharing of germplasm and knowledge—advances the Convention’s goals. When financial gain or hoarding takes precedence over collaboration and innovation, governance undermines these objectives.

### Foundation: Merton’s Norm of Disinterestedness.

Collective benefits are advanced through a commitment to impartiality, rejecting narrow personal, institutional, or national self-interests.

## Principle 6: Constructive Accountability over Fear and Stagnation

Policies and attitudes that encourage scientific skepticism and criticism of governance further the Convention’s aims. Those that foster fear of liability or reprisals fail its purpose.

### Foundation: Merton’s Norm of Organized Skepticism.

This norm recognizes that all progress starts with disagreement. Cultures and policies that celebrate open critique and rigorous questioning foster scientific and societal progress.

## Principle 7: Empiricism over Intentions

Governance should be judged by its empirically observed outcomes rather than its stated intentions. If policies fail to deliver measurable benefits for biodiversity and society, they undermine the Convention’s aims.

### Foundation: Empiricism.

Evaluations should be based on observable evidence, a principle succinctly expressed in the motto of the world’s oldest scientific society, the Royal Society: *Nullius in verba*—“*take nobody’s word for it.*”^‡‡^ As the Society explains, “*It is an expression of the determination of Fellows to withstand the domination of authority and to verify all statements by an appeal to facts.*”^§§^ (48). This principle underpins all the others: without empiricism, no reforms can be reliably advanced.

The paradox is that these principles, once articulated, may seem self-evident—yet current biodiversity governance rarely measures up to them. Their usefulness becomes clear when the most significant response to the Convention—the Plant Treaty—is measured against them. Its Multilateral System of Facilitated Exchange modestly advanced biodiversity governance with respect to Principles 2^¶¶^, 3, 4, and 5 but with respect to the others, it had no significant impact. Moreover, the modest gains were confined to the agricultural germplasm listed in Annex I. Expanding the Annex to include more crops and wild relatives would deliver further incremental improvements and, institutionally, would be the easiest route for change. But only more fundamental reform could lead to governance measuring well against all principles.

More fundamental reforms guided by the principles would draw on time-tested foundational values shared by science and good governance, with a proven record of benefit to humanity. Effective governance will need different frameworks to achieve different aims: protecting and using biodiversity in natural habitats is different to collecting germplasm and conserving it in germplasm banks for future use. Across contexts, principle-based reforms offer a compass for real-world solutions. Governance that fails the test of the principles is inconsistent with the Convention’s goals—and therefore lacks standing to claim its international recognition and protections. Acknowledging this would set clear standards and encourage approaches grounded in what works. Yet, the principles depend on shared commitments. In an international context marked by divergent cultures and competing interests, those conditions rarely arise on their own. Tailored models, along with flexibility and incentives, will be needed.

Among the possible approaches, we highlight one framework that has been developed through extensive economic analysis (49). It offers a way to align interests and foster conditions for the principles to take effect. This framework focuses on areas rich in biodiversity—hotspots—offering a model where nature would be both conserved and used, and humanity would benefit. The next section gives a brief overview of this rationale and how this could work in practice.

## Incentives for the Road Ahead

Despite aiming to encourage both the sustainable use of biodiversity and its conservation, the Convention on Biological Diversity fails to achieve much of either. Rather than providing incentives for countries to enable access to biodiversity for the global good––including for research related to agricultural development and food security—the regime has restricted access by raising transaction costs (In economics, “transaction costs” refers not just to financial costs but also to time, effort, and labor imposed by bureaucracy.). For agricultural germplasm, the Plant Treaty was designed to reduce these bureaucratic burdens and negotiations, but as noted earlier, its impact has been limited. These costs arise from the focus of both the Convention and its associated Nagoya Protocol on benefit-sharing, and they routinely occur—even for materials covered by the Treaty—because access usually requires institutional and national sign-offs, indigenous or local permissions, and other approvals, each of which entails negotiation. Moreover, as biodiversity is distributed spatially according to Nature’s rules, not national boundaries, access may require agreements involving multiple countries. By promoting a benefit-sharing principle in which each country can claim exclusive rights to the commercial value of its biodiversity, the current governance effectively creates a tragedy of the “anti-commons,” whereby overlap of ownership rights to a resource leads to a breakdown of cooperation, a disappearance of wealth, and, ultimately, wasteful and inefficient underuse of the resource (50). Because the financial returns to a specific piece of germplasm can be very small (34, 35), significant transaction costs may mean that the material is not used. And, yet, if the material is not used, the incentive to conserve it will be lost. The situation created by these agreements is a mirror image of Garrett Hardin’s Tragedy of the Commons which describes the overuse of resources held in common by multiple parties. In the governance of the Convention, countries are so preoccupied with negotiations to secure and maximize their share of the pie that no pie actually gets baked in the first place. Furthermore, by constraining scientific advancements in critical areas such as food security, this may backfire on biodiverse countries—the very countries that the Convention was intended to help (51).

Under current arrangements, countries that harbor genetic resources have an incentive to extract as much rent as they can from their assets. By contrast, these same countries can claim just a small fraction of the global benefit of Vavilov’s strategy. The incentives created by the Convention thus undermine the global interest. Where biodiversity is threatened by development, conservation imposes real financial burdens and opportunity costs. The tragedy of the anti-commons makes it hard for these countries to gain from conservation, but doing away with benefit-sharing will not, by itself, improve this incentive. For these reasons, a proposal to make germplasm available to all countries will require international financing for biodiversity conservation.

One model for reconciling access and conservation involves a mutually beneficial framework in which nations allow access to their germplasm while continuing to exercise sovereign stewardship of their biodiversity. In partnership, the international community shares the costs of conservation, including the opportunity costs of foregone development. Biodiversity “hotspots”—areas unusually rich in endemic species and unique ecosystems—would be acknowledged as national resources of global importance with their conservation supported through international financing (49). Parties would agree which sites to protect, how to protect them, and what the costs are. Financing could follow the United Nations scale of assessments, a progressive formula already used for common commitments, with the largest and wealthiest countries contributing the most. This arrangement, that could be established under a new protocol to the Convention on Biological Diversity, gives biodiverse countries reliable support for protection and sustainable use, while enabling germplasm exchange once more. By widening access, innovation and benefits multiply, flowing globally, including back to provider nations. Like a diversified portfolio, maintaining broad genetic resources reduces risk and increases the chances of finding solutions to agricultural challenges as they emerge. This framework, with its incentive structure, respects national sovereignty while aligning the interests of all parties. Biodiversity host countries would gain reliable financial support for conservation that also contributes to their development. All countries would gain wider access to genetic materials needed for innovation and improvement of the crops on which they rely. Vavilov’s strategy could work again as originally envisioned, delivering global gains in food security for all countries while, at the same time, ensuring the long-term conservation of biodiversity.

## Concluding Section

Governance that prioritizes process over progress and control over collaboration—however well-intentioned—has obstructed advances in biodiversity conservation, research and development, and food security, while delivering little benefit to biodiversity or society. In doing so, it has harmed those it aimed to protect. A shift toward enabling positive outcomes, rather than overemphasizing the prevention of negatives^##^, is urgently needed. Without reform, stagnation will persist, governance will remain entangled, unable to meet the challenges of our time, leaving a vacuum that may be filled by worldviews that disregard biodiversity and human welfare.

These principles are an invitation to all policymakers, researchers, conservationists, and the public to prioritize real-world outcomes and embrace reforms that foster collaboration and progress—thus enabling Vavilov’s ingenious strategy of improving crops to feed humanity. We hope these principles, together with governance where incentives are aligned, can help reduce hunger, foster peace, and conserve the world’s irreplaceable biodiversity.

## Supplementary Material

Appendix 01 (PDF)

## Data Availability

There are no data underlying this work.

## Footnotes

^*^Crop wild relatives are wild plant species closely related to cultivated crops, often possessing useful traits such as resistance against crop pests and diseases. They can, for instance, be used through hybridization to improve crop performance.

^†^The term germplasm refers to living plant material such as seeds, cuttings, or tissues in culture that can be used for research or crop improvement. International law usually calls the same material “genetic resources,” but we use germplasm throughout for clarity.

^‡^The temporary loan of herbarium specimens—pressed and preserved plant parts, that are not alive or possible to propagate—was once routine among botanical scholars. This practice allowed researchers in different institutions to compare specimens from various regions and confidently describe new species.

^§^The Benefit-Sharing Fund is the Plant Treaty’s fund that collects payments from the use of germplasm in its Multilateral System and distributes them for projects supporting conservation and sustainable use.

^¶^In the original Portuguese: “*meu mundo não é da onça e do macaco, meu mundo é do homem e do gado.*”

^#^This refers to the practice of cutting forest lianas in the area to be cleared and leaving them to dry; the extra dry fuel hanging in the forest canopy makes burning possible.

^||^Merton formalized and integrated these norms into a modern context; he did not create them. They were foundational preconditions for the development of science and were already expressed in the works of Plato.

^**^The recognition of biodiversity’s intrinsic value has some limited exceptions, such as for parasites like the Guinea Worm, where control or eradication aligns with broader ecological or humanitarian goals.

^††^This principle governs ease of access, conditions of access could potentially differ, for instance, for commercial versus humanitarian use.

^‡‡^*Nullius in verba* expresses the core of science: that knowledge must rest on observation. Though chosen by the Royal Society as its motto, the idea is far older. For example, a phrase attributed to the Greek sage Myson of Chenae (~600 BC)—“*We should not investigate facts by the light of arguments, but arguments by the light of facts”*—is a wonderfully pithy expression of the same principle. Empiricism was not invented by science; it was a prerequisite for its emergence.

^§§^The original wording continues *“…determined by experiment*.” We truncate it here because in modern English “experiment” is understood narrowly as a controlled test, whereas in the 17th century its use was broader and was interchangeable with “experience”, or in modern language, empirical observation.

^¶¶^Although the Plant Treaty is an extra layer of governance, it allows, to a certain extent, the bypassing of more complex prior rules.

^##^This reflects both empirical observations and philosophical critiques, such as Ninian Smart’s analysis of negative utilitarianism, which warns that overzealous harm prevention leads, in fact, to unintended harm.

## References

[1] N. E. Borlaug, The Green Revolution, Peace, and Humanity. Nobel Peace Prize Acceptance Speech (1970), https://www.nobelprize.org/prizes/peace/1970/borlaug/lecture/ [Accessed 6 Jan 2024].

[2] H. Ritchie, P. Rosado, M. Roser, Hunger and undernourishment. OurWorldInData.org (2023), https://ourworldindata.org/hunger-and-undernourishment [Accessed 8 July 2024].

[3] H. Ritchie , Population growth. Data adapted from Gapminder, PBL Netherlands Environmental Assessment Agency, United Nations. OurWorldInData.org (2023), https://ourworldindata.org/grapher/population-regions-with-projections [Accessed 8 July 2024].

[4] J. Diamond, Evolution, consequences and future of plant and animal domestication. Nature **418**, 700–707 (2002). 12167878 10.1038/nature01019

[5] J. F. N. I. Crow, Vavilov, martyr to genetic truth. Genetics **134**, 1 (1993). 8514123 10.1093/genetics/134.1.1 PMC1205417

[6] J. Janick, Nikolai Ivanovich Vavilov: Plant geographer, geneticist, martyr of science. HortScience **50**, 772–776 (2015).

[7] G. M. Volk, P. Byrne, Crop wild relatives in genebanks. Electronic Publication (2020), https://colostate.pressbooks.pub/cropwildrelatives/chapter/crop-wild-relatives-in-genebanks/.

[8] R. P. Singh , Emergence and spread of new races of wheat stem rust fungus: Continued threat to food security and prospects of genetic control. Phytopathology **105**, 872–884 (2015). 26120730 10.1094/PHYTO-01-15-0030-FI

[9] C. A. Gilligan, Developing predictive models and early warning systems for invading pathogens: Wheat rusts. Annu. Rev. Phytopathol. **62**, 217–241 (2024). 38857540 10.1146/annurev-phyto-121423-041956

[10] D. Hoisington , Plant genetic resources: What can they contribute toward increased crop productivity? Proc. Natl. Acad. Sci. U.S.A. **96**, 5937–5943 (1999). 10339521 10.1073/pnas.96.11.5937 PMC34209

[11] FAOSTAT. https://www.fao.org/faostat/ [Accessed 8 July 2024].

[12] M. L. Warburton , Bringing wild relatives back into the family: Recovering genetic diversity in CIMMYT improved wheat germplasm. Euphytica **149**, 289–301 (2006).

[13] Y. Chai, P. G. Pardey, K. A. Silverstein, Scientific selection: A century of increasing crop varietal diversity in US wheat. Proc. Natl. Acad. Sci. U.S.A. **119**, e2210773119 (2022). 36512494 10.1073/pnas.2210773119 PMC9907116

[14] R. Hajjar, T. Hodgkin, The use of wild relatives in crop improvement: A survey of developments over the last 20 years. Euphytica **156**, 1–13 (2007).

[15] M. D. Wolfe , Historical introgressions from a wild relative of modern cassava improved important traits and may be under balancing selection. Genetics **213**, 1237–1253 (2019). 31624088 10.1534/genetics.119.302757 PMC6893375

[16] D. J. Bertioli , Legacy genetics of *Arachis cardenasii* in the peanut crop shows the profound benefits of international seed exchange. Proc. Natl. Acad. Sci. U.S.A. **118**, e2104899118 (2021). 34518223 10.1073/pnas.2104899118 PMC8463892

[17] L. B. Andrews, Genes and patent policy: Rethinking intellectual property rights. Nat. Rev. Genet. **3**, 803–808 (2002). 12360238 10.1038/nrg909

[18] P. Gepts, Who owns biodiversity, and how should the owners be compensated? Plant Physiol. **134**, 1295–1307 (2004). 15084724 10.1104/pp.103.038885 PMC419806

[19] J. S. Sherkow, H. T. Greely, The history of patenting genetic material. Annu. Rev. Genet. **49**, 161–182 (2015). 26442843 10.1146/annurev-genet-112414-054731

[20] F. A. Bockmann , Brazil’s government attacks biodiversity. Science **360**, 865 (2018). 10.1126/science.aat7540 29798874

[21] Å. Bjørnstad, S. Tekle, M. Göransson, “Facilitated access” to plant genetic resources: Does it work? Genet. Resour. Crop Evol. **60**, 1959–1965 (2013).

[22] K. D. Prathapan , When the cure kills—CBD limits biodiversity research. Science **360**, 1405–1406 (2018). 29954970 10.1126/science.aat9844

[23] C. Anderson, NIH drops bid for gene patents. Science **263**, 909–910 (1994). 8310287 10.1126/science.8310287

[24] J. Fox, Sequencing, patenting surge. Nat. Biotech. **17**, 1148 (1999). 10.1038/70664 10585683

[25] G. Kolata, Celera to Charge Other Companies to Use Its Genome Data (The New York Times, 2000). 12159840

[26] European Patent Office, Revocation of European Patent No. EP0436257B1 (2000), https://register.epo.org/application?number=EP90250319 [Accessed 11 March 2025].

[27] United States Patent and Trademark Office, Reexamination Certificate (3650th), U.S. Patent No. 5,401,504 (1998), https://patentimages.storage.googleapis.com/c5/4a/0f/bd0d3ab5478eaa/US5401504.pdf [Accessed 11 March 2025].

[28] A. Browne, India fights basmati rice patent. The Guardian (2000).

[29] United States Patent and Trademark Office, Reexamination Certificate (4525th), U.S. Patent No. US 5,663,484 (2002), https://patentimages.storage.googleapis.com/a0/17/e0/f2a482dd8c81aa/US5663484.pdf [Accessed 11 March 2025].

[30] S. India-U, Fight on Basmati Rice Is Mostly Settled (Hinduism Today, 2001).

[31] United States Patent and Trademark Office, Ex parte POD-NERS, L.L.C., Appeal 2007-3938, Reexamination Control 90/005,892, Reissue Application 09/773,303, Patent 5,894,079 (2008), https://www.uspto.gov/sites/default/files/ip/boards/bpai/decisions/inform/fd073938.pdf [Accessed 11 March 2025].

[32] S. S. Dhillion , Bioprospecting: Effects on environment and development. Ambio **31**, 491–493 (2002). 12436849 10.1579/0044-7447-31.6.491

[33] E. Pennisi, Costa Rica’s INBio facing government bailout. Science (2013), https://www.science.org/content/article/costa-ricas-inbio-facing-government-bailout.

[34] R. Simpson, R.A. Sedjo. David, John W. Reid, Valuing biodiversity for use in pharmaceutical research. J. Polit. Econ. **104**, 163–185 (1996).

[35] C. Costello, M. Ward, Search, bioprospecting and biodiversity conservation. J. Environ. Econ. Manage. **52**, 615–626 (2006).

[36] I. L. Noriega , Cgiar operations under the plant treaty framework. Crop Sci. **59**, 819–832 (2019).

[37] ABS Focal Point, First royalty payment to the treaty’s benefit-sharing fund. (2018), https://www.absfocalpoint.nl/en/news-5/first-royalty-payment-to-the-treatys-benefit-sharing-fund.htm [Accessed 27 August 2025].

[38] United Nations, Convention on biological diversity (2024), https://www.un.org/en/observances/biological-diversity-day/convention. 10.1016/s0378-8741(96)90036-7 9213623

[39] H. Dempewolf , Past and future use of wild relatives in crop breeding. Crop Sci. **57**, 1070–1082 (2017).

[40] J. Gaffney , Open access to genetic sequence data maximizes value to scientists, farmers, and society. Glob. Food Secur. **26**, 100411 (2020).

[41] J. Haidt, The Righteous Mind: Why Good People Are Divided by Politics and Religion. (Pantheon/Random House, New York, NY, 2012), p. 419.

[42] J. S. Mill, On. Liberty, Dover Thrift Editions: Philosophy. (Dover Publications, Mineola, NY, [1859] 2002).

[43] Munn, Excerpted from Introduction to Psychology (Houghton-Mifflin, Boston, 1951), https://www2.nau.edu/~mid/edr610/class/research/conclusions/horsebacon.html [Accessed 8 July 2024].

[44] R. K. Merton, Science and the social order. Philos. Sci. **5**, 321–337 (1938).

[45] R. K. Merton, The normative structure of science (2024), https://www.panarchy.org/merton/science.html [Accessed 11 November 2024].

[46] K. Popper, The Open Society and Its Enemies (George Routledge & Sons, London, 1945).

[47] J. Rauch, The Constitution of Knowledge: A Defense of Truth (Brookings Institution Press, 2021).

[48] Royal Society, History of the royal society, (2024), https://royalsociety.org/about-us/who-we-are/history/.

[49] S. Barrett, A biodiversity hotspots treaty: The road not taken. Environ. Resour. Econ. **83**, 937–954 (2022).

[50] M. Heller, The tragedy of the anticommons: A concise introduction and lexicon. Mod. L. Rev. **76**, 12000 (2013).

[51] A. Deplazes-Zemp , The Nagoya protocol could backfire on the Global South. Nat. Ecol. Evol. **2**, 917–919 (2018), 10.1038/s41559-018-0561-z. 29760439

