# Supplementary material for: Reviving Vavilov’s vision: The tragedy of biodiversity governance and principles for reform
Source: Proc Natl Acad Sci U S A. 2025 Dec 12;122(51):e2501753122. doi: 10.1073/pnas.2501753122 (PMC12745805; doi:10.1073/pnas.2501753122)

Supplementary Material 1

The following Supplementary Material is a letter from the Organizing Committee of the Workshop of Germplasm Curators of Brazil in 2011 to the Minister of State for the Environment, communicating a Motion passed by the 181 Brazilian scientists and curators attending the Workshop.

Pages 2-4 are the letter and Motion translated into English.
Pages 5-7 are copies of the original document in Portuguese.

WORKSHOP OF CURATORS OF GERMPLASM OF BRAZIL
Agronomic Institute of Campinas - IAC - Campinas - SP - July 4-6, 2011
- workshop.curadores.2011@gmail.com

C. Workshop of Curators of Germplasm of Brazil n.01/2011

Brasília, August 1, 2011

To Her Excellency
IZABELLA MÔNICA VIEIRA TEIXEIRA
Minister of State for the Environment
Brasília, DF

Madam Minister,

From the 4th to the 8th of July, 2011, in Campinas, São Paulo State, the Workshop of Curators of Germplasm of Brazil was held, and as a result, a Motion was drafted addressing the legal challenges to the conservation, characterization, and utilization of germplasm of native species and their potential consequences, such as the reduction in the capacity to aggregate value to these species. We hereby submit this document for your consideration.

Respectfully,

Marília Lobo Burle
Supervisor of the Management Unit of the Curatorship System
Embrapa Genetic Resources and Biotechnology

Renato Ferraz de Arruda Veiga
President of the Coordination Committee of Curatorship
São Paulo Agency of Agribusiness Technology - APTA

Organizing Committee of the Workshop

WORKSHOP OF CURATORS OF GERMPLASM OF BRAZIL
Agronomic Institute of Campinas - IAC - Campinas - SP - July 4-6, 2011
- workshop.curadores.2011@gmail.com

MOTION OF THE WORKSHOP OF CURATORS OF GERMPLASM OF BRAZIL 2011 ON THE LEGAL DIFFICULTIES OF CONSERVATION, CHARACTERIZATION, AND UTILIZATION OF GERMPLASM OF NATIVE SPECIES AND THE POTENTIAL CONSEQUENCES OF REDUCING THE CAPACITY TO AGREGATE VALUE TO THESE SPECIES.

Considering that adding value to Brazilian biodiversity involves not only its conservation but also the availability and use of samples of its component species, enabling their proper characterization, evaluation, and demonstration of biological and economic potential,

- that the curators of germplasm banks from national and state systems and Brazilian universities are effective government agents responsible for transforming the potential to add value to germplasm into reality,

- that the complex and confusing conceptualization and application of the convoluted national legislation have created an atmosphere of uncertainty, insecurity, and fear for professionals, whose work, funded by the National Treasury and State Treasuries, involves the handling and the collection of native species,

- that this atmosphere, by potentially curbing the ability of germplasm curators and scientists to work properly, has reduced the interest in working with native species,

- that, while this continues, as it already has for more than a decade, the ecosystems of our country continue to be devastated, with loss of species and even extinction

- and that, as challenges in using native species for economic purposes continue to persist, national agricultural activities are increasingly dominated by exotic species, whose expansion often threatens natural ecosystems,

The participants of the Workshop of Curators of Germplasm of Brazil, held in Campinas – SP, in July 2011, recommend and request that:

1. Legislation on access to Brazilian genetic heritage is adjusted to reality, shifting from its fundamentally restrictive and punitive character to a role that stimulates the aggregation of value to biodiversity through its sustainable use. This includes making a clear public call to action for curators and for programs for conservation and

characterization of germplasm, respectively as agents and effective instruments of this process of aggregating value,

2. And that, until suitable changes are made, a moratorium should be established on the execution of activities related to the collection, *ex situ* conservation, characterization, evaluation, and provision of native genetic resources by researchers contracted by government agencies at the federal, state, and municipal levels.

cc:
His Excellency, Minister of State Wagner Gonçalves Rossi, Ministry of Agriculture, Livestock, and Food Supply

His Excellency, Minister of State Aloizio Mercadante Oliva, Ministry of Science and Technology

Dr. Eliana Maria G. Fontes, Director of the Department of Genetic Heritage - MMA

Dr. Maurício Lopes, Executive Director of R&D at Embrapa

Dr. Evair V. de Melo, President of the National Council of State Systems for Agricultural Research - Consepa

Dr. Orlando Melo de Castro, Coordinator of the São Paulo Agency for Agribusiness Technology - APTA

Dr. Hamilton Humberto Ramos, General Director of the Agronomic Institute - IAC

Dr. Mauro Carneiro, General Head of Embrapa Genetic Resources and Biotechnology

Dr. Messias Gonzaga Pereira, President of the Brazilian Society of Plant Breeding

Dr. Clara Oliveira Goedert, President of the Brazilian Society of Genetic Resources

WORKSHOP DE CURADORES DE GERMOPLASMA DO BRASIL 2011

INSTITUTO AGRONÔMICO DE CAMPINAS - IAC - CAMPINAS - SP - 4 A 6 DE JULHO DE 2011 - workshop.curadores.2011@gmail.com

C. Workshop de Curadores de Germoplasma do Brasil n.01/2011

Brasília, 1 de agosto de 2011

A Sua Excelência a Senhora
IZABELLA MÔNICA VIEIRA TEIXEIRA
Ministra de Estado do Meio Ambiente
Brasília, DF

Senhora Ministra,

Durante o período de 04 a 08 de julho de 2011, em Campinas/SP, realizou-se o Workshop de Curadores de Germoplasma do Brasil, e como resultado foi elaborada a Moção sobre as dificuldades legais para a execução da conservação, caracterização e uso de germoplasma de espécies nativas e suas consequências potenciais de redução da capacidade instalada para agregação de valor a tais espécies, que ora encaminhamos para apreciação.

Atenciosamente,

Marília Lobo Burle
Supervisora do Núcleo de Gestão
do Sistema de Curadorias
Embrapa Recursos Genéticos
e Biotecnologia

Renato Ferraz de Arruda Veiga
Presidente da Comissão de
Coordenação das Curadorias da
Agência Paulista de Tecnologia
dos Agronegócios - APTA

Comissão Organizadora do Workshop

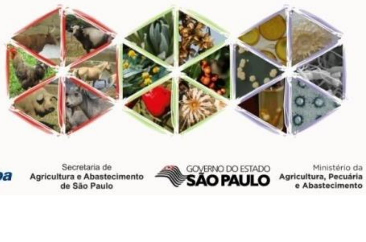

MOÇÃO DO WORKSHOP DE CURADORES DE GERMOPLASMA DO BRASIL 2011 SOBRE AS DIFICULDADES LEGAIS PARA EXECUÇÃO DA CONSERVAÇÃO, CARACTERIZAÇÃO E USO DE GERMOPLASMA DE ESPÉCIES NATIVAS E SUAS CONSEQUËNCIAS POTENCIAIS DE REDUÇÃO DA CAPACIDADE INSTALADA PARA AGREGAÇÃO DE VALOR A TAIS ESPÉCIES.

Considerando que a agregação de valor à biodiversidade brasileira implica, além de sua conservação, a disponibilidade e manipulação de amostras de suas espécies componentes, que permita sua devida caracterização, avaliação e demonstração do potencial biológico e econômico,

- que os curadores dos bancos de germoplasma dos sistemas nacional e estaduais e de universidades brasileiras são os agentes de governo efetivamente responsáveis pela transformação do potencial de agregação de valor em realidade,

- que a conceituação e aplicação confusas da intrincada legislação nacional tem criado uma atmosfera de incerteza, insegurança e amedrontamento dos profissionais, cujo trabalho, remunerado pelo Tesouro Nacional e Tesouros Estaduais, envolve a manipulação e mesmo a coleta de espécies nativas,

- que esta atmosfera, pelo potencial de cercear a liberdade de atuação dos agentes citados, vem reduzindo o interesse dos atuais e potenciais envolvidos em se dedicarem a espécies nativas,

- que, enquanto esse processo se alonga, já por mais de uma década, os ecossistemas nacionais continuam sendo devastados de modo significante, inclusive sendo depauperadas ou extintas as fontes de diversidade biológica e genética com potencial de agregação de valor econômico,

- e que, enquanto permanecem as dificuldades para colocação de espécies autóctones em uso econômico, as atividades agrárias nacionais são cada vez mais dominadas por espécies exóticas, cuja expansão muitas vezes se estende sobre ecossistemas naturais,

os participantes do Workshop de Curadores de Germoplasma do Brasil, ocorrido em Campinas – SP, em Julho de 20011, recomendam e solicitam que:

1. Seja a legislação sobre o acesso ao patrimônio genético brasileiro ajustada à realidade, deixando o atual caráter fundamentalmente restritivo e passando a ter caráter estimulador da agregação de valor à biodiversidade a seu uso sustentável, inclusive deixando manifesto o apoio concreto à ação dos curadores e dos programas de conservação e

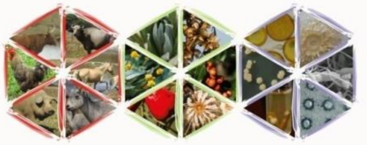

e caracterização de germoplasma, respectivamente agentes e instrumentos efetivos deste processo de agregação de valor,

2. E que, até que tal alteração se consolide, seja estabelecida moratória quanto à execução das atividades relativas à coleta, conservação *ex situ*, caracterização, avaliação e disponibilização para uso de recursos genéticos nativos por pesquisadores contratados por órgãos governamentais de nível federal, estadual e municipal.

cc.
Exmo. Sr. Ministro de Estado Wagner Gonçalves Rossi, Ministério da Agricultura, da Pecuária e do Abastecimento

Exmo. Sr. Ministro de Estado Aloizio Mercadante Oliva, Ministério da Ciência e Tecnologia

Dra. Eliana Maria G. Fontes, Diretora do Departamento de Patrimônio Genético - MMA

Dr. Maurício Lopes, Diretor-Executivo de P&D da Embrapa

Dr. Evair V. de Melo, Presidente do Conselho Nacional dos Sistemas Estaduais de Pesquisa Agropecuária - Consepa

Dr. Orlando Melo de Castro, Coordenador da Agência Paulista de Tecnologia dos Agronegócios - APTA

Dr. Hamilton Humberto Ramos, Diretor Geral do Instituo Agronômico - IAC.

Dr. Mauro Carneiro, Chefe Geral da Embrapa Recursos Genéticos e Biotecnologia.

Dr. Messias Gonzaga Pereira, Presidente da Sociedade Brasileira de Melhoramento de Plantas.

Dra. Clara Oliveira Goedert, Presidente da Sociedade Brasileira de Recursos Genéticos.

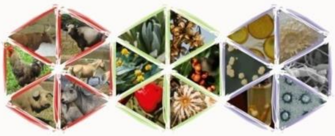

Supplement: Supplementary file 1 — Appendix 01 (PDF) [file pnas.2501753122.sapp.pdf]
